# Supplementary material for: Effectiveness of a Brief Lifestyle Intervention in the Prenatal Care Setting to Prevent Excessive Gestational Weight Gain and Improve Maternal and Infant Health Outcomes
Source: Int J Environ Res Public Health. 2022 May 11;19(10):5863. doi: 10.3390/ijerph19105863 (PMC9141141; doi:10.3390/ijerph19105863)
Supplement: Supplementary file 1 [file ijerph-19-05863-s001.zip › ijerph-1673079-supplementary.pdf]

Table S1

Results of the sensitivity analysis for the primary outcome: complete case GEE-Model

	Adj. Effect Size (95 % CI)	Adjusted p value
Women exceeding GWG recommendations	0.78	0.047
BMI < 18.5 kg/m^2	2.16	0.210
BMI 18.5–24.9 kg/m^2	0.73	0.049
BMI 25.0–29.9 kg/m^2	0.82	0.514
BMI ≥ 30.0 kg/m^2	0.88	0.629
Total gestational weight gain	-0.93	0.003
BMI < 18.5 kg/m^2	0.13	0.791
BMI 18.5–24.9 kg/m^2	-0.83	0.014
BMI 25.0–29.9 kg/m^2	-1.75	> 0.001
BMI ≥ 30.0 kg/m^2	-0.38	0.722

Results of the sensitivity analysis for the primary outcome: IPTW GEE-Model

	Adj. Effect Size (95 % CI)	Adjusted p value
Women exceeding GWG recommendations	0.78	0.030
BMI < 18.5 kg/m^2	1.38	0.588
BMI 18.5–24.9 kg/m^2	0.72	0.036
BMI 25.0–29.9 kg/m^2	0.86	0.631
BMI ≥ 30.0 kg/m^2	0.91	0.743
Total gestational weight gain	-0.89	0.003
BMI < 18.5 kg/m^2	0.09	0.811
BMI 18.5–24.9 kg/m^2	-0.82	0.026
BMI 25.0–29.9 kg/m^2	-1.53	0.002
BMI ≥ 30.0 kg/m^2	-0.51	0.595
